# Supplementary material for: Disappearing cities on US coasts
Source: Nature. 2024 Mar 6;627(8002):108–15. doi: 10.1038/s41586-024-07038-3 (PMC10917664; doi:10.1038/s41586-024-07038-3)
Supplement: Supplementary file 1 — Supplementary Figs 1–6 and legends for Supplementary Tables 1–25. [file 41586_2024_7038_MOESM1_ESM.pdf]

nature portfolio

https://doi.org/10.1038/s41586-024-07038-3

Supplementary information

Disappearing cities on US coasts

In the format provided by the
authors and unedited

Disappearing Cities on US Coasts

Leonard O. Ohenhen, Manoochehr Shirzaei, Chandrakanta Ojha, Sonam, F. Sherpa, Robert J. Nicholls

Correspondence to: ohleonard@vt.edu

The supplementary materials contain 6 figures and 25 tables:

Fig. 1. Sea-level change due to contributions from global processes. Comparison of geocentric sea-level change from the Intergovernmental Panel on Climate Change (IPCC) Sixth Assessment Report17, 34, with sea-level change due to land subsidence from Interferometric synthetic aperture radar (InSAR) measurements at 20 IPCC tide gauge stations. Atlantic Coast: (a) Boston, Massachusetts (MA); (b) Woods Hole, MA; (c) Bridgeport, Connecticut (CT); (d) The Battery, New York (NY); (e) Atlantic City, New Jersey (NJ); (f) Cape May, NJ; (g) Lewes, Delaware (DE); (h) Sewells Point, Virginia (VA); (i) Beaufort, North Carolina (NC); (j) Wilmington, NC; (k) Charleston I, South Carolina (SC); (l) Trident Pier, Florida (FL) Gulf Coast: (m) Fort Myers, FL; (n) Panama City, FL; (o) Freeport, Texas (TX); (p) Rockport, TX. Pacific Coast: (q) San Diego, California (CA); (r) Monterey, CA; (s) San Francisco, CA; (t) Crescent City, CA. The solid pink lines shows the median (50th percentile) IPCC sea-level change under shared socioeconomic pathways (SSP) scenario SSP1-1.9 (low emission scenario), while the pink shaded range shows the 17th – 83rd percentile. The solid brown line shows the median (50th percentile) IPCC sea-level change under SSP3-7.0 (medium to high emission scenario), while the brown shaded range shows the 17th – 83rd percentile. The solid blue line shows the InSAR vertical land motion from this study, and the blue-shaded ranges are 1 standard deviation.

Fig. 2. Influence of glacial isostatic adjustment (GIA) on vertical land motion (VLM) across the US coast. (a) GIA data across the US from the ICE-6G-D model70. (b) VLM for the US coasts without GIA effect (Background Image: Google, Earthstar). National and state boundaries in (a) and (b) are based on public domain vector data by World DataBank (https://data.worldbank.org/). (c) Empirical cumulative distribution function (CDF) for total VLM and VLM without GIA effect for the US Atlantic coast. (d) Empirical CDF for total VLM and VLM without GIA effect for the US Gulf coast. (e) Empirical CDF for total VLM and VLM without GIA effect for the US Pacific coast. State Codes: MA Massachusetts, NY New York, NJ New Jersey, MD Maryland, VA Virginia, NC North Carolina, SC South Carolina, GA Georgia, FL Florida, AL Alabama, MS Mississippi, LA Louisiana, TX Texas, and CA California.

Fig. 3. Distribution of glacial isostatic adjustment (GIA) effects on vertical land motion (VLM) for selected US coastal cities. (a) Boxplots representing the distribution of total VLM and VLM without GIA for 11 US Atlantic coastal cities. (b) Boxplots representing the distribution of total VLM and VLM without GIA for 11 US Gulf coastal cities. (c) Boxplots representing the distribution of total VLM and VLM without GIA for 11 US Pacific coastal cities.

Fig. 4. Flowchart describing the research methodology. (a) Multitemporal interferometric synthetic aperture radar (InSAR) processing using Wavelet-Based InSAR (WabInSAR) algorithm56–60. (b) Combination of InSAR and global navigation satellite system (GNSS) datasets to generate 3D velocities. (c) Static inundation modeling employed in this study. LOS is line-of-sight velocity, VLM is vertical land motion, LiDAR DEM is Light detection and ranging digital elevation model, IPCC is the Intergovernmental Panel on Climate Change, SLR is sea level rise, NOAA is the National Oceanic and Atmospheric Administration, and MHW is mean high water.

Fig. 5. Vertical land motion (VLM) error and validation analysis. (a) VLM standard deviation distribution map for the US Atlantic, Gulf, and Pacific coasts (Background Image: Google, Earthstar). National and state boundaries in (a) are based on public domain vector data by World DataBank (https://data.worldbank.org/). Histogram comparing global navigation satellite system (GNSS) vertical rates with interferometric synthetic aperture radar (InSAR) VLM rates for (b) US Pacific coast, (c) US Atlantic coast, and (d) US Gulf coast. MN is the mean difference between GNSS and InSAR rates. SD is the standard deviation of the difference between GNSS and InSAR rates. N is the number of GNSS station. A subset of the GNSS stations are shown in Extended Data Figs. 2 to 4. State Codes: MA Massachusetts, NY New York, NJ New Jersey, MD Maryland, VA Virginia, NC North Carolina, SC South Carolina, GA Georgia, FL Florida, AL Alabama, MS Mississippi, LA Louisiana, TX Texas, and CA California.

Fig. 6. Percent area exposure for census blocks by 2050. Histogram of percent area exposure for census blocks in (a) Boston, (c) Biloxi, (e) Richmond. Note that census blocks without any area exposed are not shown here. The dashed black line and adjacent value represent the median percent exposure for each city. Probability density function for (b) Boston, (d) Biloxi, (f) Richmond.

Table 1. Total population, properties, and home value for US cities. This contains the population, properties, and, home value for the 32 cities analyzed in this study. The population and properties are estimated by calculating the sum of the population and properties for the census blocks in each city. The home value for each city is estimated using the zip-code level Zillow Home Value Index (ZHVI).

Table 2. Modeled exposed area, population, properties, and home value for cities on the US Atlantic Coast by 2050. a, b, and c represent the lower bound, median value, and upper bound of exposure estimated using Equation 3, respectively.

Table 3. Modeled exposed area, population, properties, and home value for cities on the US Gulf Coast by 2050. a, b, and c represent the lower bound, median value, and upper bound of exposure estimated using Equation 3, respectively.

Table 4. Modeled exposed area, population, properties, and home value for cities on the US Pacific Coast by 2050. a, b, and c represent the lower bound, median value, and upper bound of exposure estimated using Equation 3, respectively.

Table 5. Contribution of vertical land motion (VLM) and relative sea level rise (SLR) for US Atlantic coast. Estimated exposure for cities considering VLM only and VLM and SLR. This considers all projected elevation below sea level (i.e. 0 m), hydrologic connection to the coast is not considered here. a, b, and c represent the lower bound, median value, and upper bound of exposure estimated considering VLM standard deviation and error from digital elevation model, respectively.

Table 6. Contribution of vertical land motion (VLM) and relative sea level rise (SLR) for US Gulf coast. Estimated exposure for cities considering VLM only and VLM and SLR. This considers all projected elevation below sea level (i.e. 0 m), hydrologic connection to the coast is not considered here. a, b, and c represent the lower bound, median value, and upper bound of exposure estimated considering VLM standard deviation and error from digital elevation model, respectively.

Table 7. Contribution of vertical land motion (VLM) and relative sea level rise (SLR) for US Pacific coast. Estimated exposure for cities considering VLM only and VLM and SLR. This considers all projected elevation below sea level (i.e. 0 m), hydrologic connection to the coast is not considered here. a, b, and c represent the lower bound, median value, and upper bound of exposure estimated considering VLM standard deviation and error from digital elevation model, respectively.

Table 8. Comparison of InSAR and IPCC derived exposure for US Atlantic coast. InSAR (Interferometric Synthetic Aperture Radar) derived exposure is estimated using InSAR vertical land motion (VLM) and IPCC SLR (Intergovernmental Panel on Climate Change)30 as detailed in the methods section, while the IPCC derived exposure is estimated using IPCC relative SLR projections30. a, b, and c represent the lower bound, median value, and upper bound of exposure estimated using Equation 3, respectively.

Table 9. Comparison of InSAR and IPCC derived exposure for US Gulf coast. InSAR (Interferometric Synthetic Aperture Radar) derived exposure is estimated using InSAR vertical land motion (VLM) and IPCC SLR (Intergovernmental Panel on Climate Change)30 as detailed in the methods section, while the IPCC derived exposure is estimated using IPCC relative SLR projections30. a, b, and c represent the lower bound, median value, and upper bound of exposure estimated using Equation 3, respectively.

Table 10. Comparison of InSAR and IPCC derived exposure for US Pacific coast. Interferometric Synthetic Aperture Radar (InSAR) derived exposure is estimated using InSAR vertical land motion (VLM) and Intergovernmental Panel on Climate Change (IPCC) SLR 30 as detailed in the methods section, while the IPCC derived exposure is estimated using IPCC relative SLR projections30. a, b, and c represent the lower bound, median value, and upper bound of exposure estimated using Equation 3, respectively.

Table 11. Comparison of InSAR and IPCC vertical land motion (VLM) rates (mm per year) for the US coast. Interferometric Synthetic Aperture Radar (InSAR) VLM rates are median rates and standard deviation for cities derived from this study, while the Intergovernmental Panel on Climate Change (IPCC) VLM rates are 17th, 50th, and 83rd percentile of VLM rates from Garner et al.30.

Table 12. Comparison of InSAR and IPCC vertical land motion (VLM) rates (mm per year) at tide gauge stations across the US coast. Interferometric Synthetic Aperture Radar (InSAR) VLM rates are the average VLM rates of InSAR pixels within a 200 m radius of the tide gauges and the standard deviation associated with each InSAR pixel. Intergovernmental Panel on Climate Change (IPCC) VLM rates are 17th, 50th, and 83rd percentile of VLM rates obtained from Garner et al.30. A statistical z-test is used to test for differences between the VLM rates and standard deviation at each tide gauge station. The null hypothesis for the z-test is that there is no statistical difference between rates of VLM. p-value for z-test is shown in the table colored green when we accept the null hypothesis and red where we reject the null hypothesis at alpha = 0.05.

Table 13. Total population versus exposed population by 2050 for different racial demographics on the US Atlantic Coast. The distribution of the total and exposed population is expressed as both the population count and as a percentage of the population. Minority groups include individuals identifying as Black or African American, American Indian or Alaska Native, Asian, Native Hawaiian or Other Pacific Islander, Hispanic or Latino, other races, and two or more groups. a, b, and c represent the lower bound, median value, and upper bound of exposure, respectively.

Table 14. Total population versus exposed population by 2050 for different racial demographics on the US Gulf Coast. The distribution of the total and exposed population is expressed as both the population count and as a percentage of the population. Minority groups include individuals identifying as Black or African American, American Indian or Alaska Native, Asian, Native Hawaiian or Other Pacific Islander, Hispanic or Latino, other races, and two or more groups. a, b, and c represent the lower bound, median value, and upper bound of exposure, respectively.

Table 15. Total population versus exposed population by 2050 for different racial demographics on the US Pacific Coast. The distribution of the total and exposed population is expressed as both the population count and as a percentage of the population. Minority groups include individuals identifying as Black or African American, American Indian or Alaska Native, Asian, Native Hawaiian or Other Pacific Islander, Hispanic or Latino, other races, and two or more groups. a, b, and c represent the lower bound, median value, and upper bound of exposure, respectively.

Table 16. Distribution of the total properties value versus the value of exposed properties by 2050 for cities on the US Atlantic coast. A statistical t-test is used to test for differences between the total property value in each city and the exposed property value. The null hypothesis for the t-test is that there is no statistical difference between the value of the total properties in the city and the value of the exposed properties. p-value for t-test is shown in the table colored

green when we reject the null hypothesis and red where we accept the null hypothesis at alpha = 0.05. a, b, and c represent the lower bound, median value, and upper bound of exposure, respectively.

Table 17. Distribution of the total properties value versus the value of exposed properties by 2050 for cities on the US Gulf coast. A statistical t-test is used to test for differences between the total property value in each city and the exposed property value. The null hypothesis for the t-test is that there is no statistical difference between the value of the total properties in the city and the value of the exposed properties. p-value for t-test is shown in the table colored green when we reject the null hypothesis and red where we accept the null hypothesis at alpha = 0.05. a, b, and c represent the lower bound, median value, and upper bound of exposure, respectively.

Table 18. Distribution of the total properties value versus the value of exposed properties by 2050 for cities on the US Pacific coast. A statistical t-test is used to test for differences between the total property value in each city and the exposed property value. The null hypothesis for the t-test is that there is no statistical difference between the value of the total properties in the city and the value of the exposed properties. p-value for t-test is shown in the table colored green when we reject the null hypothesis and red where we accept the null hypothesis at alpha = 0.05. a, b, and c represent the lower bound, median value, and upper bound of exposure, respectively.

Table 19. Distribution of levees for the thirty-two cities across the US Coast. The levee data are obtained from the US Army Corps of Engineers (USACE) (https://levees.sec.usace.army.mil/#/).

Table 20. Influence of flood control structures on exposure by 2050 for the US Atlantic Coast. This is the defended scenario for the US Atlantic coast. Note that only New York, Virginia Beach, and Miami contain at least 1 floodwall/levee on the US Atlantic coast. The percentage (%) change quantifies the change from the defended scenario to the undefended scenario presented as either a percent decrease (negative numbers) or no change (zero). a, b, and c represent the lower bound, median value, and upper bound of exposure, respectively.

Table 21. Influence of flood control structures on exposure by 2050 for the US Gulf Coast. This is the defended scenario for the US Gulf coast. Note that only New Orleans, Slidell, Port Arthur, Galveston, Texas City, Freeport, and Corpus Christi contain at least 1 floodwall/levee on the US Gulf Coast. The percentage (%) change quantifies the change from the defended scenario to the undefended scenario presented as either a percent decrease (negative numbers) or no change (zero). a, b, and c represent the lower bound, median value, and upper bound of exposure, respectively.

Table 22. Influence of flood control structures on exposure by 2050 for the US Pacific Coast. This is the defended scenario for the US Pacific coast. The percentage (%) change quantifies the change from the defended scenario to the undefended scenario presented as either a percent decrease (negative numbers) or no change (zero). a, b, and c represent the lower bound, median value, and upper bound of exposure, respectively.

Table 23. Synthetic Aperture Radar datasets.

Table 24. Coastal elevation, sea level projections, and high-tide estimates data summary for the US coastal cities. MHW indicates mean high water in meters (NAVD88).

Table 25. Property Value (US$) of different Zip Codes for cities on the US coasts. The property value is obtained from the zip-code level Zillow Home Value Index (ZHVI) (https://www.zillow.com/research/data/).

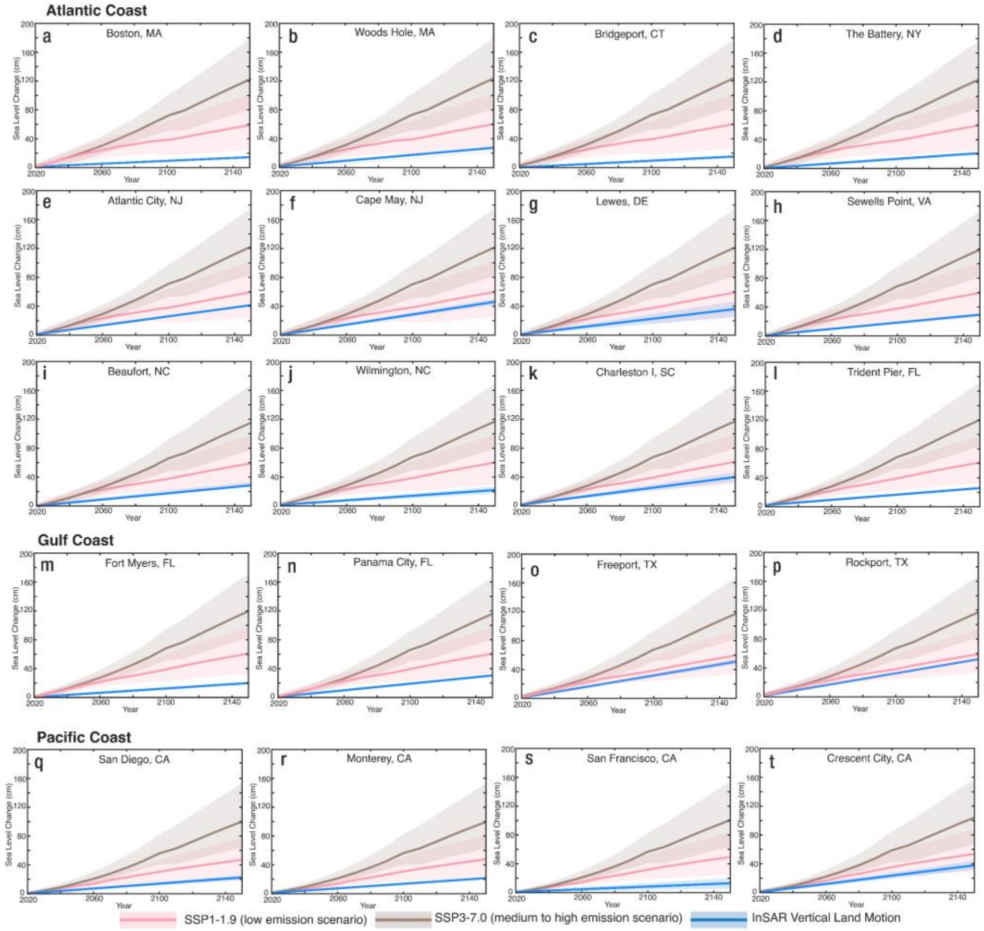

Fig. 1. Sea-level change due to contributions from global processes. Comparison of geocentric sea-level change from the Intergovernmental Panel on Climate Change (IPCC) Sixth Assessment Report[17, 34], with sea-level change due to land subsidence from Interferometric synthetic aperture radar (InSAR) measurements at 20 IPCC tide gauge stations. Atlantic Coast: **(a)** Boston, Massachusetts (MA); **(b)** Woods Hole, MA; **(c)** Bridgeport, Connecticut (CT); **(d)** The Battery, New York (NY); **(e)** Atlantic City, New Jersey (NJ); **(f)** Cape May, NJ; **(g)** Lewes, Delaware (DE); **(h)** Sewells Point, Virginia (VA); **(i)** Beaufort, North Carolina (NC); **(j)** Wilmington, NC; **(k)** Charleston I, South Carolina (SC); **(l)** Trident Pier, Florida (FL) Gulf Coast: **(m)** Fort Myers, FL; **(n)** Panama City, FL; **(o)** Freeport, Texas (TX); **(p)** Rockport, TX. Pacific Coast: **(q)** San Diego, California (CA); **(r)** Monterey, CA; **(s)** San Francisco, CA; **(t)** Crescent City, CA. The solid pink lines shows the median (50th percentile) IPCC sea-level change under shared socioeconomic pathways (SSP) scenario SSP1-1.9 (low emission scenario), while the pink shaded range shows the 17th – 83rd percentile. The solid brown line shows the median (50th percentile) IPCC sea-level change under SSP3-7.0 (medium to high emission scenario), while the brown shaded range shows the 17th – 83rd percentile. The solid blue line shows the InSAR vertical land motion from this study, and the blue-shaded ranges are 1 standard deviation.

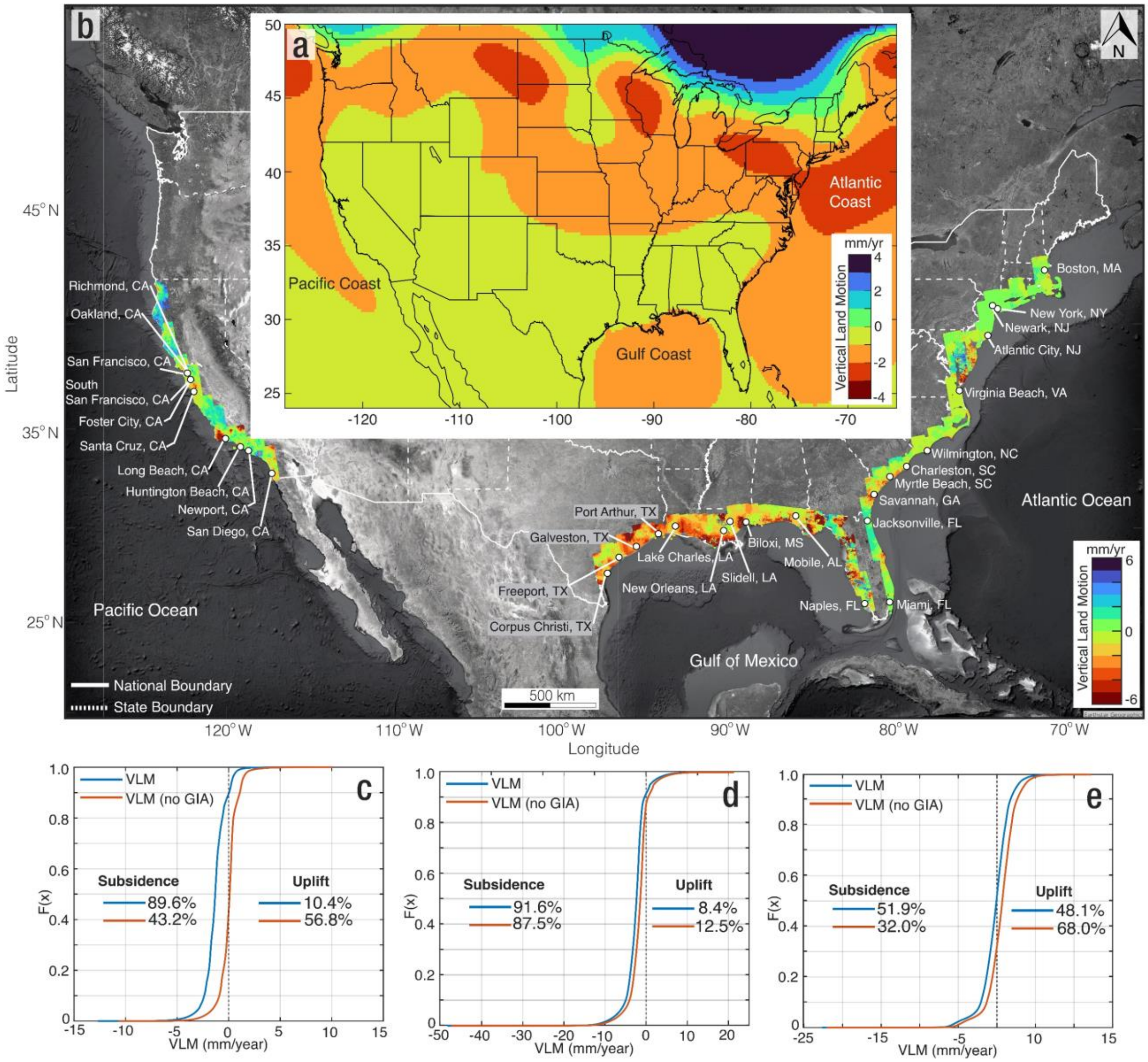

Fig. 2. Influence of glacial isostatic adjustment (GIA) on vertical land motion (VLM) across the US coast. (a) GIA data across the US from the ICE-6G-D model[70]. **(b)** VLM for the US coasts without GIA effect (Background Image: Google, Earthstar). National and state boundaries in **(a)** and **(b)** are based on public domain vector data by World DataBank (https://data.worldbank.org/). **(c)** Empirical cumulative distribution function (CDF) for total VLM and VLM without GIA effect for the US Atlantic coast. **(d)** Empirical CDF for total VLM and VLM without GIA effect for the US Gulf coast. **(e)** Empirical CDF for total VLM and VLM without GIA effect for the US Pacific coast. State Codes: MA Massachusetts, NY New York, NJ New Jersey, MD Maryland, VA Virginia, NC North Carolina, SC South Carolina, GA Georgia, FL Florida, AL Alabama, MS Mississippi, LA Louisiana, TX Texas, and CA California.

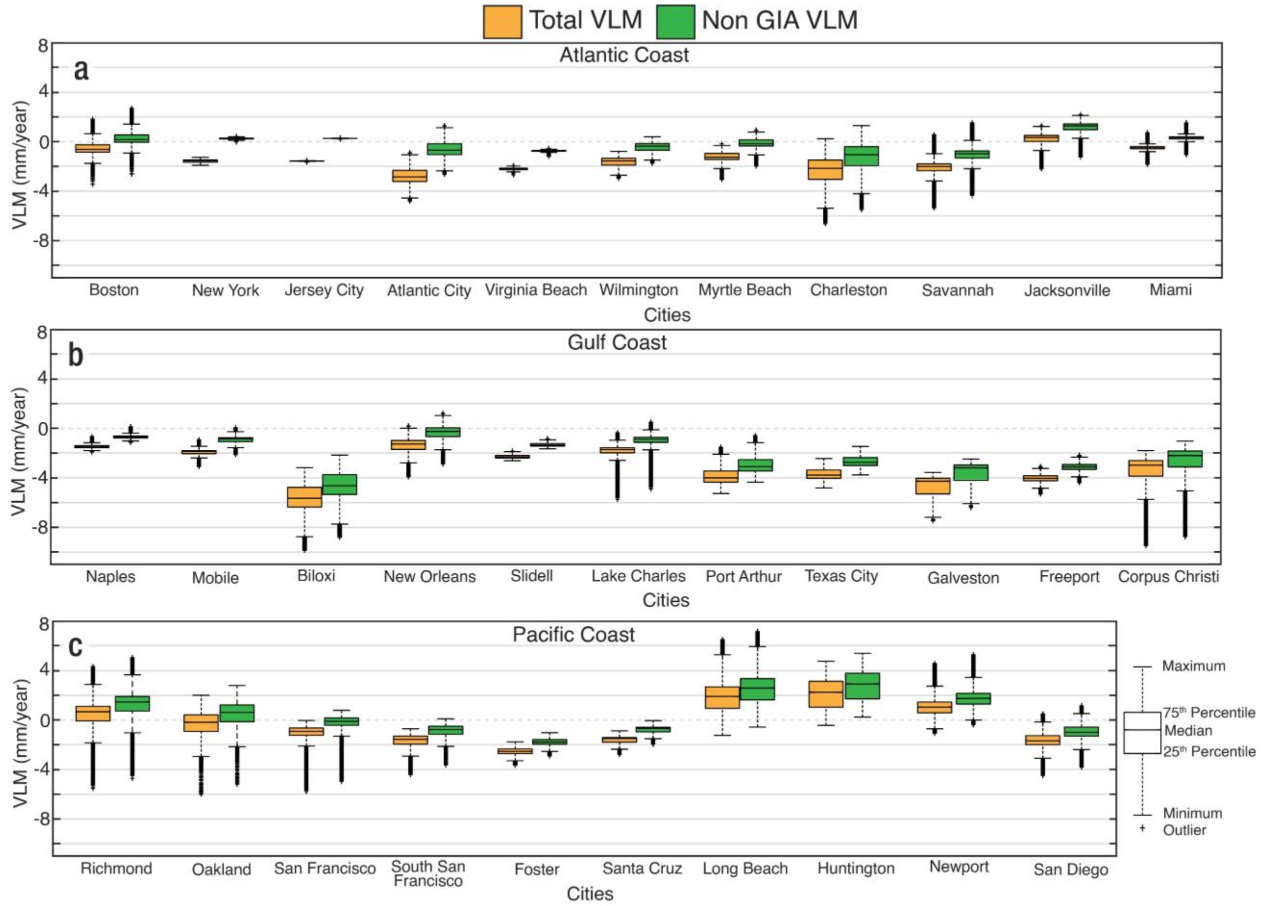

Fig. 3. Distribution of glacial isostatic adjustment (GIA) effects on vertical land motion (VLM) for selected US coastal cities. (a) Boxplots representing the distribution of total VLM and VLM without GIA for 11 US Atlantic coastal cities. **(b)** Boxplots representing the distribution of total VLM and VLM without GIA for 11 US Gulf coastal cities. **(c)** Boxplots representing the distribution of total VLM and VLM without GIA for 11 US Pacific coastal cities.

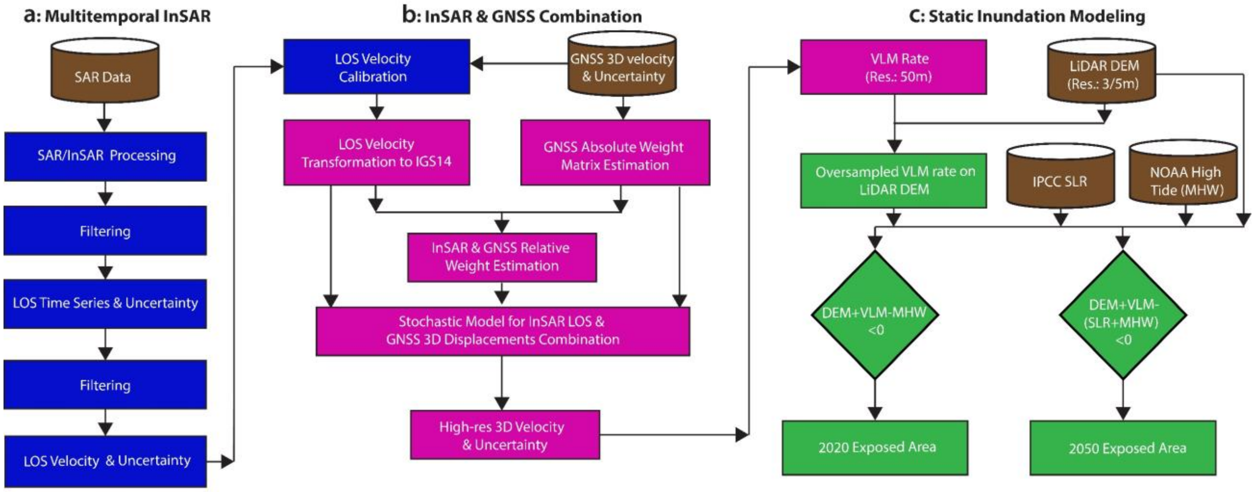

Fig. 4. Flowchart describing the research methodology. (a) Multitemporal interferometric synthetic aperture radar (InSAR) processing using Wavelet-Based InSAR (WabInSAR) algorithm[56–60]. **(b)** Combination of InSAR and global navigation satellite system (GNSS) datasets to generate 3D velocities. **(c)** Static inundation modeling employed in this study. LOS is line-of-sight velocity, VLM is vertical land motion, LiDAR DEM is Light detection and ranging digital elevation model, IPCC is the Intergovernmental Panel on Climate Change, SLR is sea level rise, NOAA is the National Oceanic and Atmospheric Administration, and MHW is mean high water.

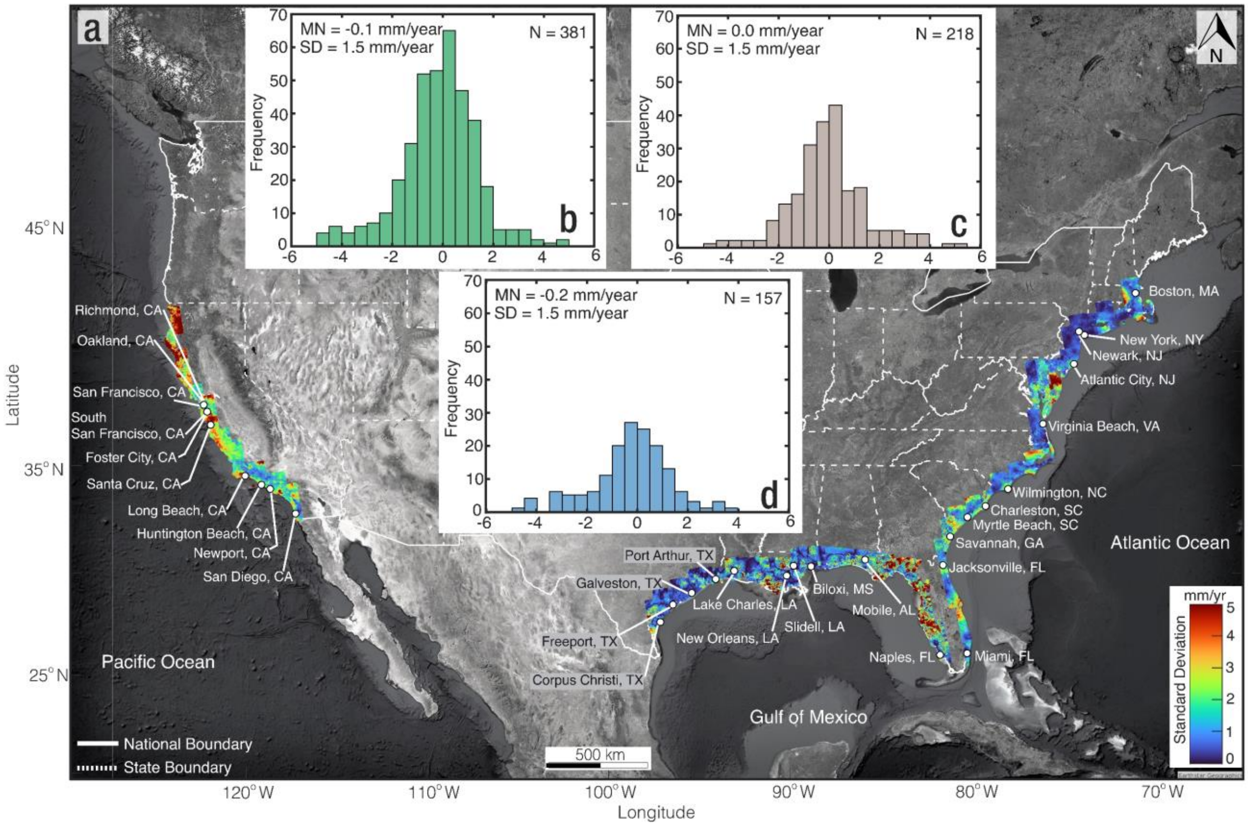

Fig. 5. Vertical land motion (VLM) error and validation analysis. (a) VLM standard deviation distribution map for the US Atlantic, Gulf, and Pacific coasts (Background Image: Google, Earthstar). National and state boundaries in **(a)** are based on public domain vector data by World DataBank (https://data.worldbank.org/). Histogram comparing global navigation satellite system (GNSS) vertical rates with interferometric synthetic aperture radar (InSAR) VLM rates for **(b)** US Pacific coast, **(c)** US Atlantic coast, and **(d)** US Gulf coast. MN is the mean difference between GNSS and InSAR rates. SD is the standard deviation of the difference between GNSS and InSAR rates. N is the number of GNSS station. A subset of the GNSS stations are shown in Extended Data Figs. 2 to 4. State Codes: MA Massachusetts, NY New York, NJ New Jersey, MD Maryland, VA Virginia, NC North Carolina, SC South Carolina, GA Georgia, FL Florida, AL Alabama, MS Mississippi, LA Louisiana, TX Texas, and CA California.

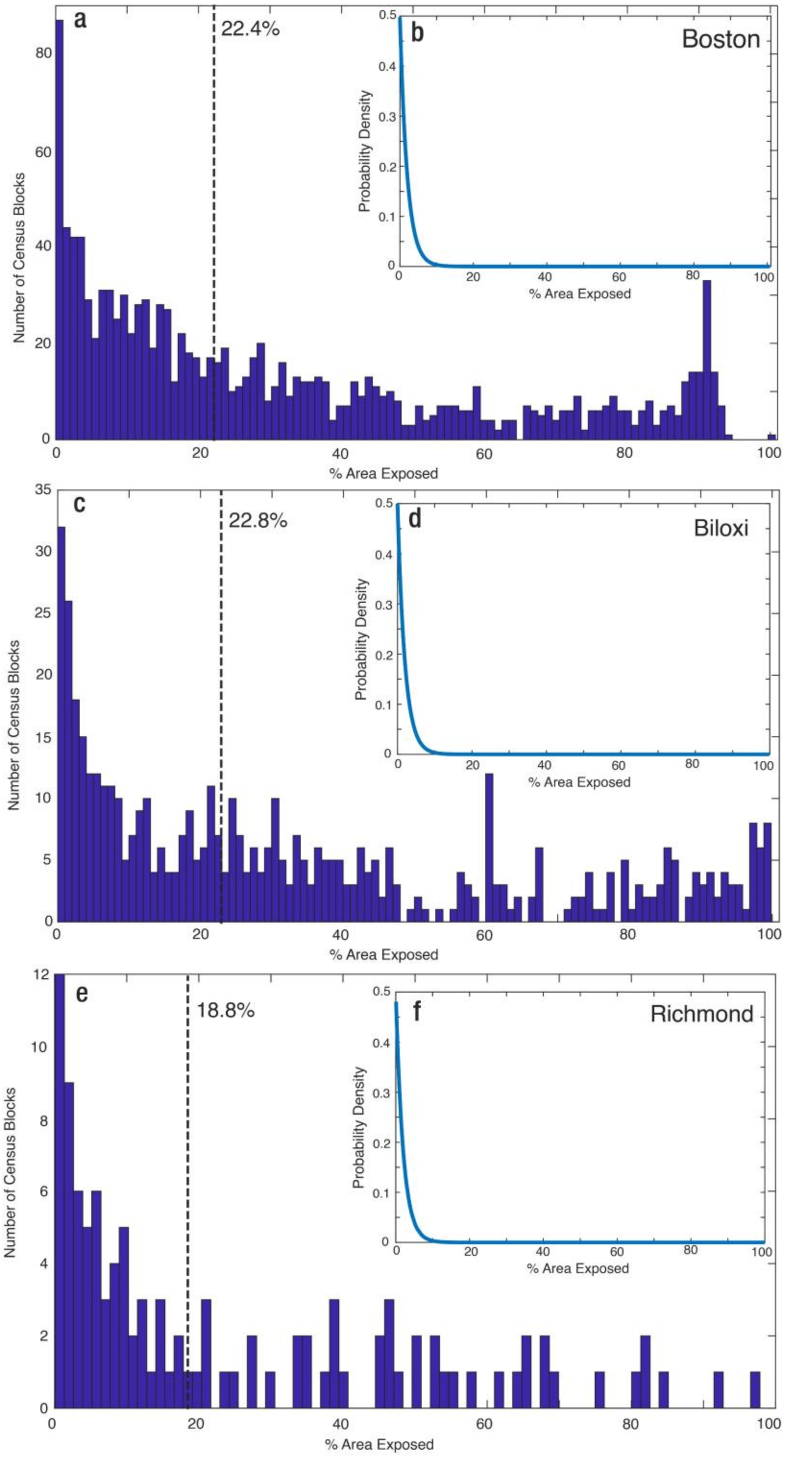

Fig. 6. Percent area exposure for census blocks by 2050. Histogram of percent area exposure for census blocks in **(a)** Boston, **(c)** Biloxi, **(e)** Richmond. Note that census blocks without any area exposed are not shown here. The dashed black line and adjacent value represent the median percent exposure for each city. Probability density function for **(b)** Boston, **(d)** Biloxi, **(f)** Richmond.
